# Structural prediction of GluN3 NMDA receptors

**DOI:** 10.3389/fphys.2024.1446459

**Published:** 2024-08-20

**Authors:** Yunsheng Liu, Da Shao, Shulei Lou, Zengwei Kou

**Affiliations:** ^1^ Cancer Center, Shenzhen Hospital (Futian) of Guangzhou University of Chinese Medicine, Shenzhen, China; ^2^ Department of Neurosurgery, Institute of Translational Medicine, Shenzhen Second People’s Hospital/the First Affiliated Hospital of Shenzhen University Health Science Center, Shenzhen, China; ^3^ Research Center of Translational Medicine, Shanghai Children’s Hospital, School of Medicine, Shanghai Jiao Tong University, Shanghai, China; ^4^ Institute of Hospital Management, Linyi People’s Hospital, Linyi, China; ^5^ Department of Laboratory Medicine and Pathobiology, Temerty Faculty of Medicine, University of Toronto, Toronto, ON, Canada

**Keywords:** NMDA receptors, AlphaFold, RoseTTAFold, protein prediction, ion channel, ligand receptors

## Abstract

*N*-methyl-*D*-aspartate (NMDA) receptors are heterotetrametric ion channels composed of two obligatory GluN1 subunits and two alternative GluN2 or GluN3 subunits, forming GluN1-N2, GluN1-N3, and GluN1-N2-N3 type of NMDA receptors. Extensive research has focused on the functional and structural properties of conventional GluN1–GluN2 NMDA receptors due to their early discovery and high expression levels. However, the knowledge of unconventional GluN1-N3 NMDA receptors remains limited. In this study, we modeled the GluN1-N3A, GluN1-N3B, and GluN1-N3A-N3B NMDA receptors using deep-learned protein-language predication algorithms AlphaFold and RoseTTAFold All-Atom. We then compared these structures with GluN1-N2 and GluN1-N3A receptor cryo-EM structures and found that GluN1-N3 receptors have distinct properties in subunit arrangement, domain swap, and domain interaction. Furthermore, we predicted the agonist- or antagonist-bound structures, highlighting the key molecular–residue interactions. Our findings shed new light on the structural and functional diversity of NMDA receptors and provide a new direction for drug development. This study uses advanced AI algorithms to model GluN1-N3 NMDA receptors, revealing unique structural properties and interactions compared to conventional GluN1-N2 receptors. By highlighting key molecular–residue interactions and predicting ligand-bound structures, our research enhances the understanding of NMDA receptor diversity and offers new insights for targeted drug development.

## Introduction

*N*-methyl-*D*-aspartate receptors (NMDARs) are known as “coincidence” detectors since their activation needs both membrane depolarization to remove the Mg^2+^ blockage and binding to agonists (Traynelis et al., 2010). In the postsynaptic area, NMDAR activation allows the influx of Na^+^ and Ca^2+^, further depolarizing the membrane potential to transmit chemical signals to electric signals and initiating the Ca^2+-^/ Na^+^-related signaling pathways. Because of this, NMDARs contribute to synaptic plasticity, learning, and memory (Iacobucci and Popescu, 2017). Altered expression, subcellular distribution, or dysfunction of NMDARs are associated with neurological and psychiatric disorders (Paoletti et al., 2013).

NMDARs have seven subunits: the obligatory glycine-bound GluN1 subunit, alternative glutamate-bound GluN2 (2A, 2B, 2C, and 2D) subunits, and glycine-bound GluN3 (3A and 3B) subunits (Stroebel and Paoletti, 2021). These subunits can form diheteromeric (di-) or triheteromeric (tri-) NMDARs by two GluN1 subunits and two identical or different GluN2 or GluN3 subunits (Rauner and Kohr, 2011; Hansen et al., 2014; Bhattacharya et al., 2018; Yi et al., 2019). Unlike GluN1-N2 NMDARs, the activation of GluN1-N3 NMDARs needs only glycine binding and does not need NMDA or glutamate binding (Pfisterer et al., 2020; Bossi et al., 2022; Rouzbeh et al., 2023). Therefore, some researchers called GluN1–GluN3 “atypical” NMDARs, “unconventional” NMDARs, or excitatory glycine receptors.

By using *in situ* hybridization or antibody labeling, studies revealed that GluN3A is highly expressed in the early developmental stage, while it decreases in the adult stage (Murillo et al., 2021), and GluN3B is highly expressed in the adolescent stage and remains at a considerable expression level in the adult stage (Berg et al., 2013; Wee et al., 2016), suggesting that they may participate in various physiological processes of the brain. Recently, a pioneer study isolated the native GluN1-N3A current in the juvenile hippocampal slice for the first time by pre-incubation with CGP-78608, a glycine binding site antagonist of GluN1 (Pfisterer et al., 2020). This landmark study historically quelled the controversy on whether GluN1-N3 is an artificial receptor that only can be expressed in the heterologous expression systems and facilitated the search for their physiological function. Based on this study, further functional study revealed that astrocyte fine-tunes neuronal excitability via GluN1-N3A in the medial habenula (MHb), which is involved in emotion control in the brain (Otsu et al., 2019; Bossi et al., 2022). The current of GluN1-N3B can also be detected in the recombinant expression system (Kvist et al., 2013b; Zeng et al., 2022). This current can also be enhanced as it is in GluN1-N3A by two mutants (F484 and T517) that abolish the binding of glycine to GluN1 (Kvist et al., 2013a; Mesic et al., 2016). This suggests that the GluN1-N3B shares similar activation mechanisms with GluN1-N3A.

The structural mechanisms of GluN1-N2 NMDARs, especially GluN1-N2A and GluN1-N2B, have received much attention (Wang and Furukawa, 2019). Structural properties of GluN3-containing NMDARs remain largely unknown. Recently, the full-length structure of GluN1-N3A binding with the agonist and antagonist has been revealed by Cyro-EM (Michalski and Furukawa, 2024). However, the amino terminal domain and transmembrane domain of these receptors are not well-defined, which limits the understanding of the structural basis of these receptors. Artificial intelligence-based protein structure prediction methods, such as the AlphaFold from Google’s DeepMind (Evans et al., 2022; Abramson et al., 2024), EMF fold from Meta (Lin et al., 2023), and RoseTTAFold from David Baker’s Lab (Baek et al., 2023), can predict protein structures with high accuracy, enabling us to understand the arrangement of subunit and domain interactions of GluN1-N3 receptors. We employed AlphaFold2 and 3 to predict the structures of full-length GluN1-N3A, GluN1-N3B, and GluN1-N3A-N3B receptors. All models were tested by algorithms such as pLDDT with high overall confidence. We found that all the GluN3 NMDAR models presented a three-layer pseudo four symmetry bouquet-like shape. We, therefore, compared the predicted GluN1-N3 structures with the GluN1-N2 and GluN1-N3A receptor structures revealed by cryo-EM and found that the biggest difference between GluN1-N2 and GluN1-N3 receptors was in the arrangement of the amino terminal domain. Using RoseTTAFold All-Atom, we obtained the GluN3 models that were agonist- or antagonist-bound, which may shed light on the activation and inhibition mechanisms of these receptors.

## Results

### The expression and distribution of GluN3

**FIGURE 1:**
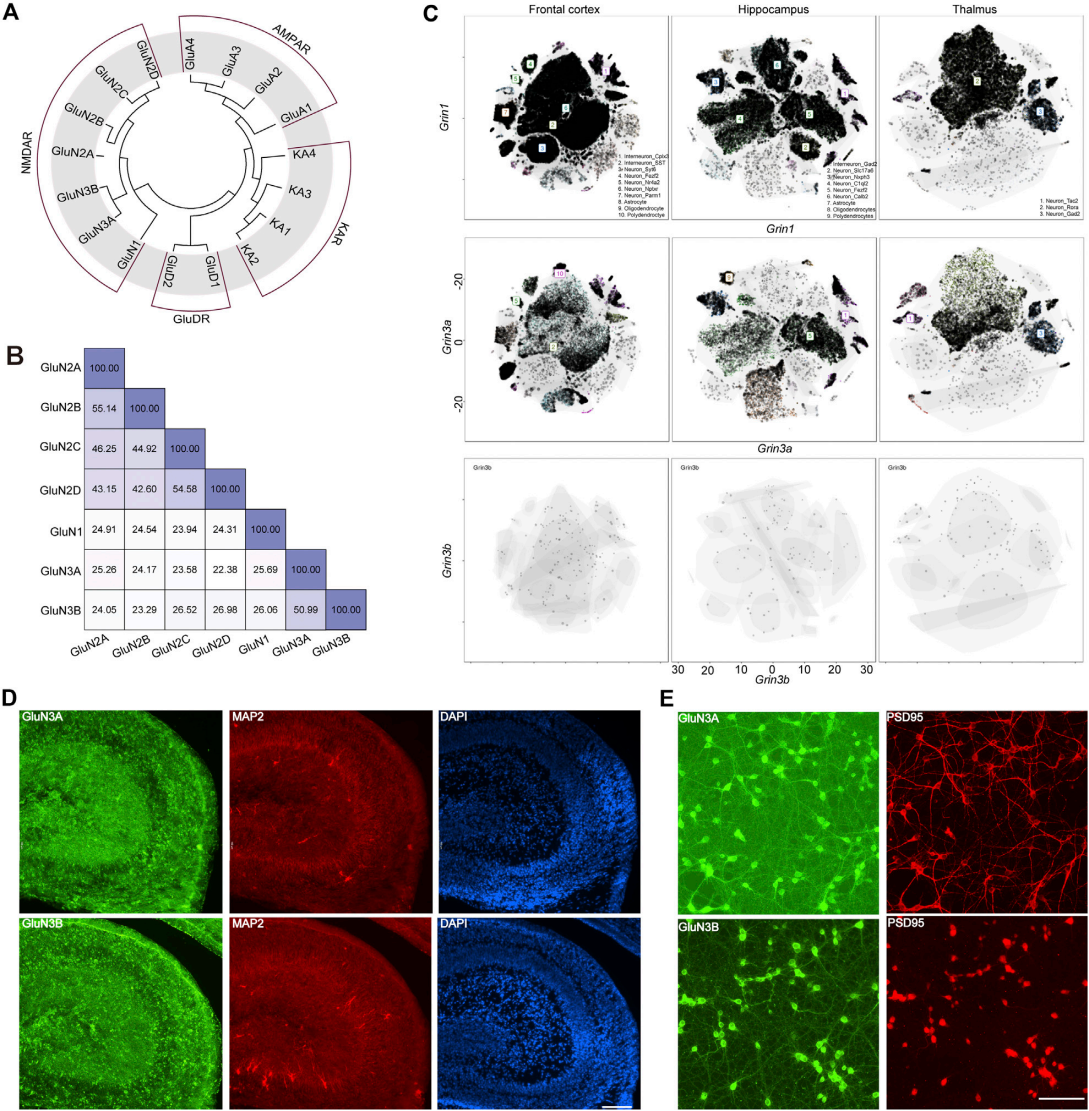
Evolutionary information, sequence alignment, and expression pattern of GluN3 subunits. **(A)** Evolutionary analysis of AMPARs, NMDARs, and KARs. **(B)** Sequence alignment results of NMDARs. **(C)** t-distributed stochastic neighbor embedding (t-SNE) plots for the frontal cortex, the hippocampus, and the thalamus global clustering of *Grin1*, *Grin3a*, and *Grin3b*. **(D)** Immunofluorescence staining of GluN3A and GluN3B in postnatal 2-day hippocampus brain slice. Scale bar = 500 μm. **(E)** Immunofluorescence staining of GluN3A and GluN3B in cultured hippocampal neurons. Scale bar = 200 μm.

GluN3A (previously known as the χ-1 subunit) and GluN3B were cloned from brain tissue in 1995 (Ciabarra et al., 1995) and 2001 (Andersson et al., 2001), respectively. In humans, the *GRIN3A* gene encoding GluN3A is located on chromosome 9 at 9q34.3, while *GRIN3B* is situated on chromosome 19 at 19q13.3. *GRIN3A* consists of 10 exons, encodes 1,115 amino acids, and shares a 51% protein sequence identity with GluN3B, which spans 901 amino acids and is encoded by nine exons in *GRIN3B*. Evolutionary analysis showed that the GluN3 family is more closely related to GluN1, with both GluN3A and GluN3B sharing 26% sequence identity with GluN1. GluN3A shows a sequence identity of 25% with GluN2A, 24% with GluN2B, 24% with GluN2C, and 22% with GluN2D, while GluN3B has sequence identities of 24%, 23%, 27%, and 27% with GluN2A, GluN2B, GluN2C, and GluN2D, respectively. On average, GluN3A exhibits a 22% sequence identity with non-NMDA ionotropic glutamate receptors, indicating that GluN3 is likely a unique subunit lineage within NMDARs evolutionarily (Figures 1A, B).

The GluN1 subunit is expressed throughout the entire brain during development, while the GluN3A and GluN3B subunits are thought to exhibit distinct spatiotemporal expression patterns (Perez-Otano et al., 2016). To gain an understanding of these three subunits in the brain, we assessed the scRNA-seq based on online datasets. We selected three brain regions, the frontal cortex, the hippocampus, and the thalamus, based on previous reports (Otsu et al., 2019; Murillo et al., 2021; Bossi et al., 2022). In general, the expression level of *Grin1* is far higher than those of *Grin3a* and *Grin3b*. These three subunits can be detected in the neurons and non-neuron neural cells. The top three cell clusters for *Grin3a* in the frontal cortex are SST-positive interneurons, Nptxr-positive neurons, and polydendrocytes. In the hippocampus, the top three cell clusters for *Grin3b* are Gad2-positive neurons, Fezf2-positive neurons, and oligodendrocytes. Only two clusters of cells can be detected in the thalamus that highly express *Grin3a*: the Tac2-positive neurons and Gad2-positive neurons. *Grin3b* can barely be detected in the above brain regions (Figure 1C), which may suggest the limited expression of *Grin3b* in the brain. To check the expression of these subunits at the protein level, we performed immunofluorescence (IF) staining in hippocampal brain slices from postnatal day 2 mice (Figure 1D) and cultured hippocampal neurons (Figure 1E). Both GluN3A and GluN3B are highly expressed in the hippocampus. Consistent with brain-slice staining results, both subunits show high expression in cultured neurons. More interestingly, the subunits are expressed in both the PSD95-positive and PSD95-negative regions, which indicates that GluN3A and GluN3B can be expressed in both pre- and post-synaptic regions (Figures 1D, E).

### Predicting the GluN1-N3A structure by AlphaFold

**FIGURE 2:**
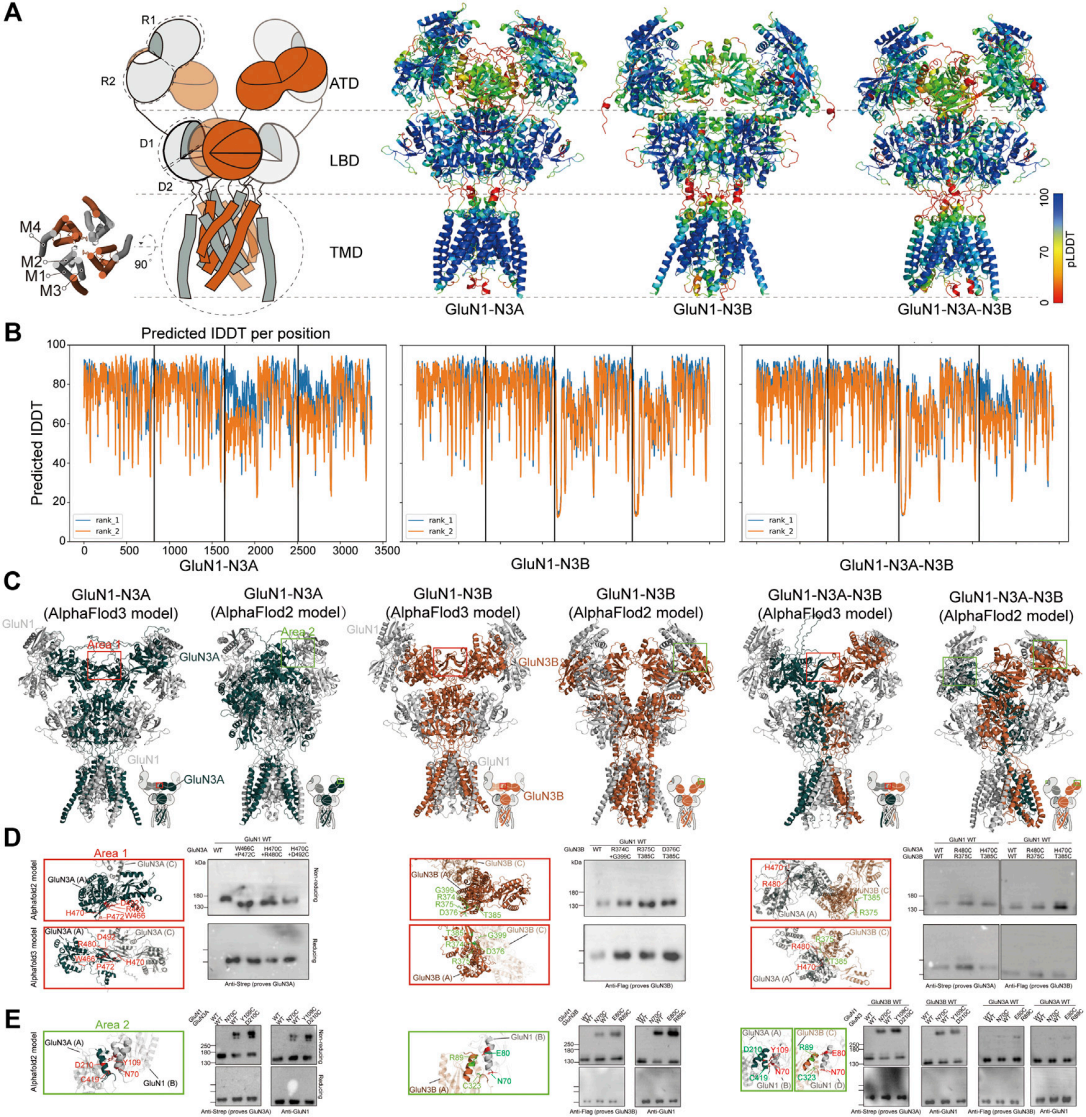
AlphaFold models and crosslinking validation of GluN1-N3A, GluN1-N3B, and GluN1-N3A-N3B receptors. **(A)** Topology and model of GluN1-N3 NMDARs. The models were colored by confidence score (pLDDT) from very low confidence (red) to good confidence (yellow) to high confidence (blue). **(B)** Plot of pLDDT confidence score versus GluN1, GluN3A, and GluN3B residue position. **(C)** Comparison of AlphaFold2 and AlphaFold3 models. Two sets of amino acids located in area 1 (marked by red rectangles) and area 2 (green rectangles) were used for cysteine mutation. **(D)** Locations of mutated residues at the ATD GluN3–GluN3 interface (Area 1) in AlphaFold2 and AlphaFold3 models and the corresponding detection of disulfide bonds by antibodies prove GluN1 and GluN3 in reducing and non-reducing conditions, respectively. **(E)** Locations of mutated residues at the ATD GluN3–GluN1 interface (area 2) in AlphaFold2 models and the corresponding detection of disulfide bonds by antibodies prove GluN1 and GluN3 in reducing and non-reducing conditions.

We used AlphaFold2 and 3 to predict the structures of GluN1-N3A, GluN1-N3B, and GluN1-N3A-N3B receptors. For the AlphaFold3 prediction, we used a seed-free pattern. For AlphaFold2, we used the ColabFold version (Mirdita et al., 2022). The ColabFold pipeline accelerated the AlphaFold2 prediction by using the MMseq2 multiple sequence alignment method instead of the Jackhmmer multiple sequence alignment method used in the original AlphaFold2 pipeline (Evans et al., 2022). We further reduced the burden on computer resources by truncating the flexible C-terminal domain (CTD) of each subunit, which is responsible for receptor trafficking and membrane anchoring, based on each subunit structure in the Protein Structure Database (Jumper et al., 2021). The protein sequences of CTD-truncated GluN1 (K24-Q847), GluN3A (P37-Q971), and GluN3B (Q24-T885) were used as input for the AlphaFold prediction. ColabFold was run as model-free, and the max_seq and max_extra_seq were set to 508 × 2,048. The recycle was set to two rounds, and amber was used for relaxation. The predicted models were qualified by the predicted local distance difference test (pLDDT) confidence score. The pLDDT score above 50 was considered acceptable. Values above 70 represent good confidence. Generally, pLDDT scores of the cores of the amino-terminal domain (ATD), ligand-binding domain (LBD), and transmembrane domain (TMD) are very high (pLDDT > 90), while the scores at the beginning of the ATD and the flexible linkers of ATD-LBD and LBD-TMD are relatively low. Of note, the flexible loop in GluN3A ATD (H46-T121) also showed a low pLDDT score. Surprisingly, AlphaFold2 predicted the transmembrane regions and pore domains, which are not well-defined in the experimental map, with good confidence (pLDDT > 90) (Figures 2A, B).

We then compared the models predicted by AlphaFold2 and AlphaFold3. In general, they all adopt a pseudo-two-fold symmetric bouquet-like structure. The models of AlphaFold2 and AlphaFold3 differ in their domain arrangement, especially in the ATD and the linkage between ATD and LBD. The AlphaFold2 models are more like conventional NMDARs, while the AlphaFold3 models exhibit some novel features not observed in conventional NMDARs (Figure 2C). Specifically, in the ATD layer, AlphaFold3 shows that the two symmetric GluN1 subunits adopt a widely open conformation, whereas GluN3 tends to lie parallel to the cell membrane, with the R2 regions of GluN3’s ATD coming closer together to form an interaction interface. In GluN1-N3A, hydrogen bonds stabilize the two GluN3A subunits’ interface through amino acids W466 and P472, H470 and R480, and H470 and D492. In GluN1-N3B, the two GluN3B subunits’ interface is stabilized by residues R374 and G399, R375 and T385, and D376 and T385. In GluN1-N3A-N3B, six pairs of hydrogen bonds are involved in maintaining the GluN3A and GluN3B interface (Figure 2D).

To investigate which structure predicted by AlphaFold2 or AlphaFold3 is more accurate, we designed crosslinking experiments. Based on the GluN1-N3A and GluN1-N3B models from AlphaFold3, we mutated the aforementioned amino acids to cysteine and performed crosslinking. The results showed that none of the pairs could form crosslinks, suggesting that these models might not represent the true structure (Figure 2D). Then, using the same strategy, we selected several amino acids from AlphaFold2 models for mutation and crosslinking validation. We found that in GluN1-N3A, the GluN1 N70C mutation could crosslink GluN1 and GluN3A, and similarly, GluN1 Y109C and GluN3A D210C could also crosslink. In GluN1-N3B, the GluN1 N70C, as well as GluN1 E80C and GluN3B R89C, mediated the crosslinking of GluN1 and GluN3B. In the system co-transfected with the three subunits, the aforementioned four pairs of mutations could also mediate the crosslinking of GluN1 and GluN3 (Figure 2E). Based on the above validation, we used the AlphaFold2 models for further comparison.

### Cross-comparison of GluN1-N3 models with cryo-EM GluN1-N2 structures

To gain more information on the structural basis of the predicted GluN1-N3 models, we conducted a comparative analysis between our models and the experimental conventional GluN1-N2 cryo-EM maps. Given that GluN1-N3A and GluN1-N3B are diheteromeric and GluN1-N3A-N3B is triheteromeric, we selected GluN1-N2A (PDB: 7EOS) (Wang et al., 2021), GluN1-N2B (PDB: 6W11) (Chou et al., 2020), and GluN1-N2A-N2B (PDB: 5UOW) (Lu et al., 2017) structures for comparison. Although they belong to the NMDAR family, they are not identical receptors and even exhibit significant sequence differences. Therefore, we focused on comparing their domain arrangements rather than helix-level or amino acid-level comparisons.

**FIGURE 3:**
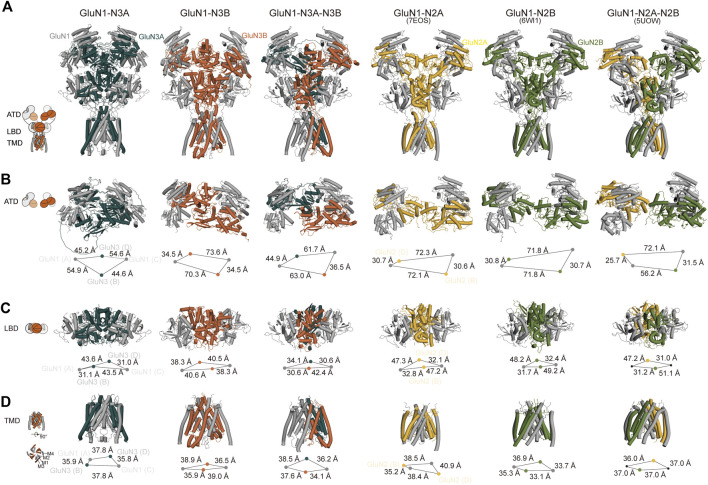
Cross-comparison of GluN1-N3 models with cryo-EM GluN1-N2 structures. AlphaFold-predicted GluN1-N3A, GluN1-N3B, and GluN1-N3A-N3B models are compared with cryo-EM-revealed GluN1-N2A (PDB: 7EOS), GluN1-N2B (PDB: 6WI1), and GluN1-N2A-N2B (PBD: 5UOW) as a whole **(A)** and at ATD **(B)**, LBD **(C)**, and TMD **(D)** layers.

Overall, all three predicted models exhibit a bouquet-like structure similar to traditional NMDARs, comprising three layers: ATD, LBD, and TMD. During the transmission process from ATD to LBD and then to TMD, the domains follow a clockwise sweep. From a top–down perspective, the ATD structures maintain a pattern where two GluN1 subunits are positioned distally, and GluN3 subunits are proximal to the assumed central axis (Figure 3A).

However, at the ATD level, unlike the traditional dimer-of-dimer combination, the two GluN1 subunits and two GluN3A subunits appear relatively independent and pseudo-four-fold symmetrical in GluN1-N3A receptors. For instance, the distances from GluN3A (B) to its adjacent GluN1 subunits (A) and (C) are similar (54.9 Å and 44.6 Å). This characteristic seems specific to GluN3A subunits, as observed in GluN1-N3A-N3B, where the distance from GluN3A to its dimeric partners (44.9 Å) is much greater than that of GluN3B subunits to their partners (36.5 Å). In contrast, GluN3B’s ATD exhibits the typical dimer-of-dimer arrangement seen in conventional NMDARs, where GluN3B (B) forms a dimer with GluN1 (C), with a distance of 35.8 Å shorter than that to the other GluN1 (A). These differences in GluN1-N2A, GluN1-N2B, and GluN1-N2A-N2B distances are 41.5 Å, 41.1 Å, and 24.7 Å (for N2A) and 46.4 Å (for N2B), respectively (Figure 3B).

At the LBD level, due to domain swapping, conventional NMDARs exhibit a dimer-of-dimer formation. Different from the A–D and B–C dimer formation at the ATD layer, the LBD layer’s dimer formation is A–B and C–D (Figure 3C). Unlike in the ATD level, the distances between LBD dimers-of-dimers are very close, resulting in a more compact domain. This compact interaction is reflected in GluN3A subunits, where the distance from GluN3A (B) to its dimer counterpart GluN1 (A) is 31.1 Å, compared to the distance to the other GluN1 subunit (C), which is 43.5 Å. GluN3B in GluN1-N3B differs from other NMDARs, with similar distances of 40.6 Å and 38.3 Å to the two GluN1 subunits. The GluN3B (B) in GluN1-N3A-N3B shows a similar pattern in conventional GluN1-N2 receptors, with a short distance (30.6 Å) to its dimer counterpart GluN1 (A) and a long distance to the other GluN1 (42.4 Å). The GluN3A (D) subunit in the GluN1-N3A-N3B receptor has relatively small differences in distances to the two GluN1 subunits, 30.6 Å and 34.1 Å, unlike its behavior in GluN1-N3A subunits. These distance differences are 14.4 Å, 17.5 Å, 19.9 Å, and 16.2 Å in GluN1-N2A, GluN1-N2B, and GluN1-N2A-N2B, respectively (Figure 3C).

At the TMD level, each subunit consists of three fully transmembrane helices (M1, M3, and M4) and a semi-transmembrane M2 helix (Figure 3D). In GluN1-N3A and GluN1-N3B, the subunit arrangement is similar to that of conventional GluN1-N2 receptors, i.e., N1–N2–N1–N2. Using the center-of-mass (COM) of the M3 helix as a reference, we observed distances of 35.9 Å and 37.8 Å from GluN3A (B) to adjacent GluN1 units and 39.0 Å and 35.9 Å for GluN3B (B). These distances are comparable to those of GluN1-N2A, GluN1-N2B, and GluN1-N2A-N2B, ranging from 35–40 Å. Notably, GluN1-N3A-N3B’s TMD subunit arrangement differs from that of other NMDARs, following an N1–N1–N3–N3 pattern. This arrangement is also present in another model of this receptor (data not shown).

### Comparison of GluN1-N3A with cryo-EM GluN1-N3A structures

The first full-length structure of the GluN1-N3A receptors was published by Michalski and Furukawa (2024), revealing both the glycine-bound “active” state and the CNQX-bound “inhibitory” state, providing us with cryo-EM structures to compare with our two AlphaFold predicted models.

**FIGURE 4:**
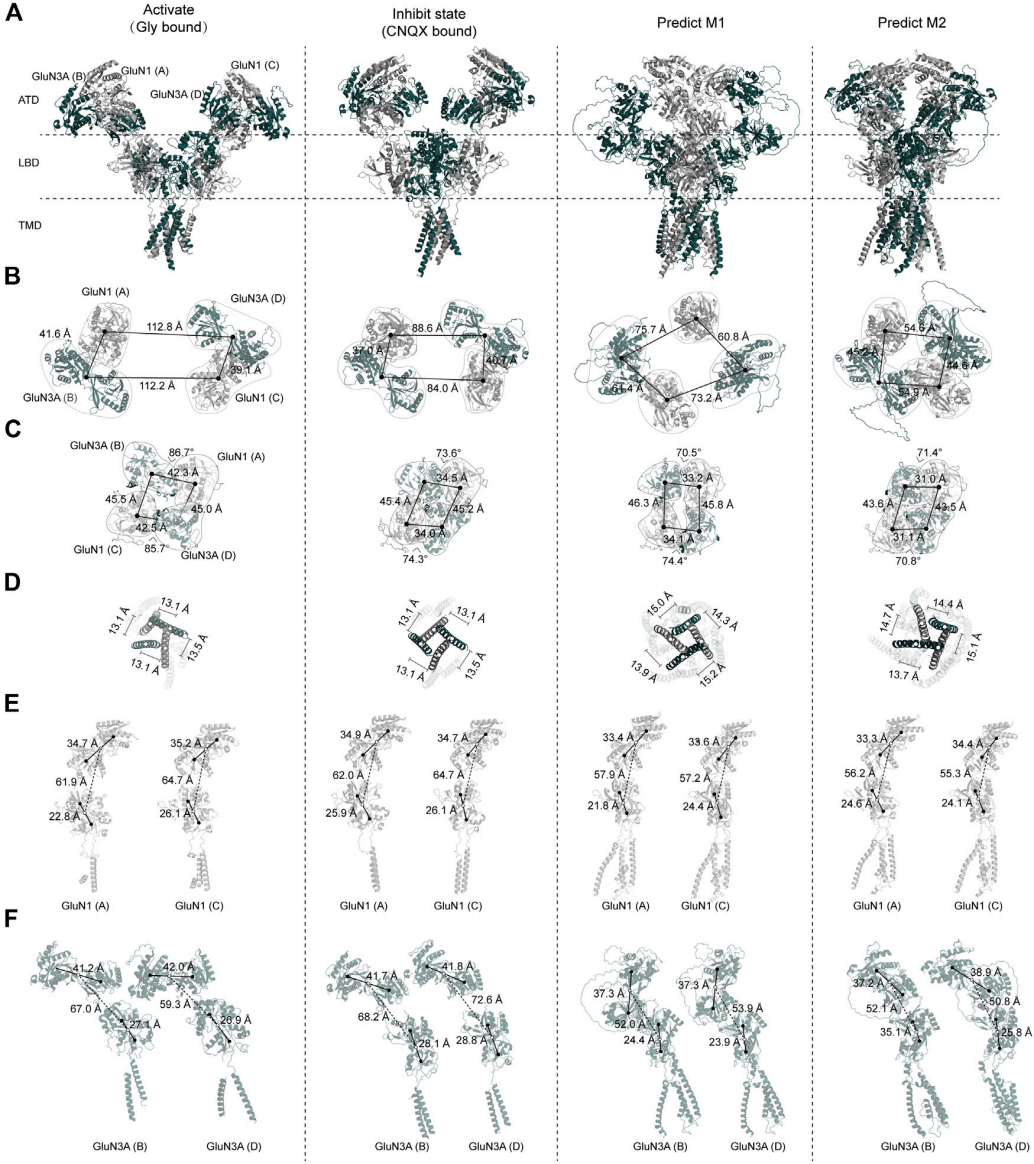
Comparison of GluN1-N3A AlphaFold models with cryo-EM structure models. **(A)** Overall structures of GluN1-N3A glycine-bound cryo-EM structures (PDB: 8USX), CNQX-bound cryo-EM structures (PDB: 8USW), and AlphaFold predicted model 1 and model 2. **(B–D)** Comparison of ATD, LBD, and TMD domains. **(E, F)** Cross-comparison of GluN1 and GluN3A subunits.

Overall, there are significant differences between the “active” and “inhibitory” states of the GluN1-N3A receptor. In the glycine-bound state, the entire receptor exhibits a wide-open and loose conformation. The distance between the ATD of the GluN1 (A) subunit and the GluN3A (D) subunit in another “dimer” is 112.8 Å. This distance is 24.2 Å longer than in the CNQX-bound state and 52.0 Å longer than in the predicted model 1. This is almost double the distance observed in model 2 (54.6 Å) (Figures 4A, B). During the transition from ATD to LBD, a clockwise domain swap occurs. In the glycine-bound structure, this clockwise domain rotation is approximately 80°. Similar to the ATD, the glycine-bound LBD is also loose, whereas the CNQX-bound LBD is tight. The tight LBD is also observed in model 1 and model 2. In the glycine-bound state, the four subunits form a pseudo-tetramer with approximately equal distances between them. In contrast, the CNQX-bound state and both models exhibit a clear dimer-of-dimer arrangement, with much shorter distances within dimers than between dimers. The total length of the LBD domains of the four subunits, from the longest to shortest, is glycine-bound > CNQX-bound > model 1 > model 2 (Figure 4C).

Due to the resolution limitations of cryo-EM for the TMD layer, we used the center of mass (COM) of the M3 helix to evaluate the pore state. We found that our AlphaFold models are “looser” than the cryo-EM structures, as evidenced by the total COM distance being significantly longer in the models than in both the CNQX-bound and glycine-bound states. Notably, the distances form a nearly equilateral quadrilateral, suggesting that each subunit may contribute significantly and equally to the pore (Figure 4D).

From this analysis, we observed that the most significant differences among the four structures are in the ATD. We further analyzed the relationship between the ATD and the ATD–LBD transition for individual subunits. Regarding the ATD, the R1–R2 distances in the glycine-bound, CNQX-bound, and both predicted structures are very similar, ranging from 33.3 to 35.2 Å. The predicted models, however, show a more compact arrangement compared to the cryo-EM structures. During the ATD–LBD transition, in the cryo-EM structures, the distance of GluN1(A) is consistently shorter than that of GluN1(C), with differences of 2.8 and 2.7 Å, respectively. In the glycine-bound state and model 1, we also observed that the LBD of GluN1(A) is more tightly packed, with the D1–D2 distance being 3.3 and 2.6 Å shorter than that of GluN1(C). Compared to the GluN1 ATD, the GluN3A ATD is more loosely arranged, a trend more pronounced in the cryo-EM structures. For instance, in the glycine-bound state, the R1–R1 distances are 41.2, 42.0, 41.7, and 41.8 Å, which are significantly longer than their corresponding GluN1 distances. In the predicted models, these distances are relatively shorter, at 37.3, 37.3, 37.2, and 38.9 Å.

We found notable differences between the two GluN1 subunits in the cryo-EM structures. In terms of the ATD–LBD interaction, the GluN3A in the glycine-bound state is more tightly packed compared to the CNQX-bound state, with lengths of 67.0 and 59.3 Å in GluN3A (A) and (C) compared to 68.2 and 72.6 Å, respectively. The predicted models are even more compact, with lengths of 52.0, 53.9, 52.1, and 50.8 Å (Figures 4E, F).

This comprehensive analysis reveals that the primary structural differences among the four states lie in the ATD, with significant implications for understanding the conformational dynamics of the GluN1-N3A receptor.

### The interactions of ATD, LBD, and TMD of the predicted models

The predicted structures provide detailed side-chain information, allowing for more comprehensive structural analyses, such as domain–domain interactions. We then analyzed the interaction sites of the dimer-of-dimer interface, including the R1–R1 interaction in the ATD and the interaction in the LBD.

**FIGURE 5:**
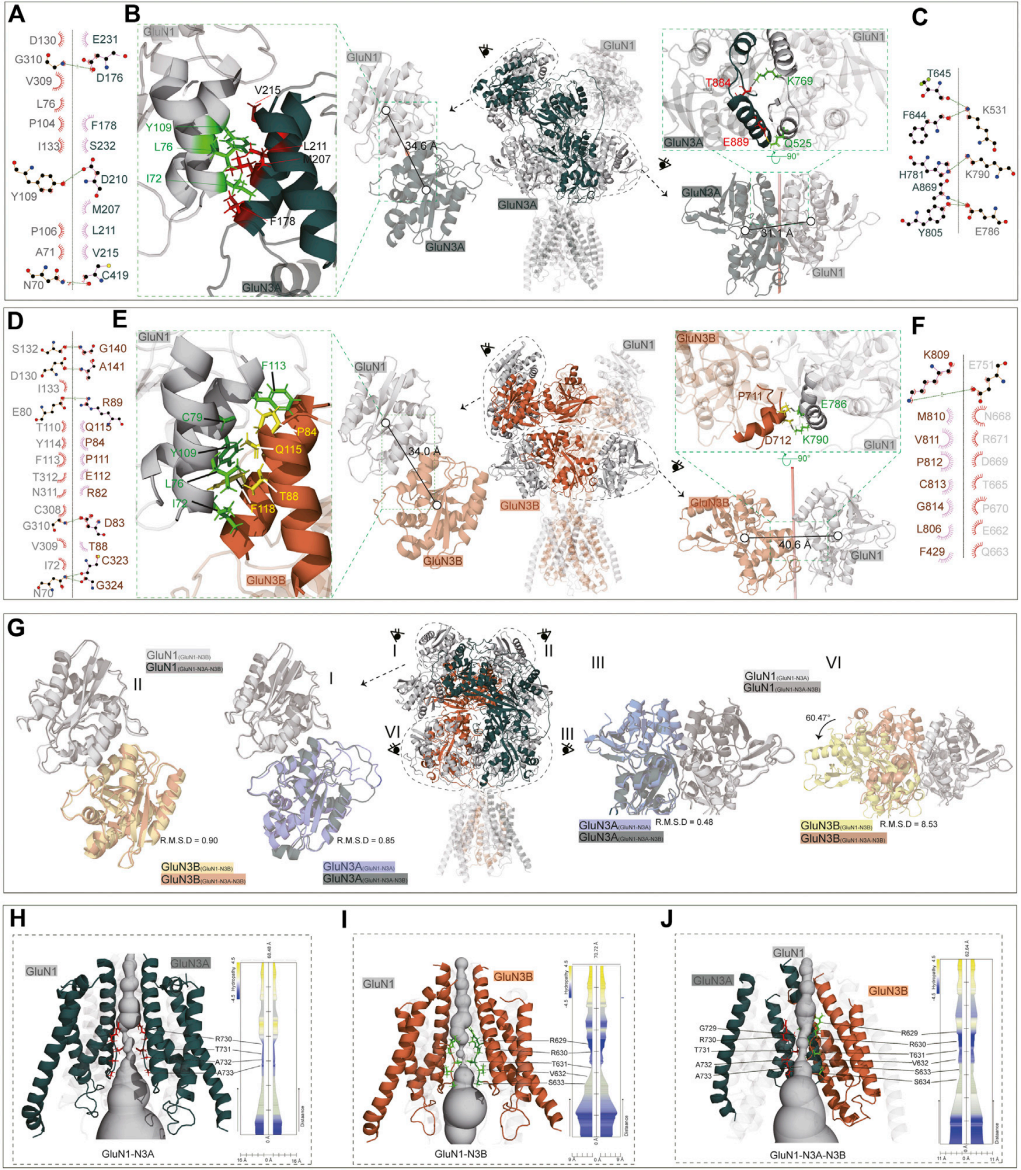
ATD, LBD interaction, and TMD domain architecture. **(A, D)** Ligplot^+^ analysis of the R1–R1 interaction of the GluN1-N3A receptor and GluN1-N3B receptor. **(B, E)** The ATD interaction and LBD interaction are indicated in the full-length receptor model by the dotted circle. The interfaces of ATD and LBD are further zoomed in the green dotted square on the left or right of the full-length receptor model. **(C, F)** Ligplot^+^ analysis of the LBD interaction of the GluN1-N3A receptor and GluN1-N3B receptor. **(G)** Comparison of ATD interaction (I, II) and LBD interaction (III, IV) of the GluN1-N3A dimer or GluN1-N3B dimer from the GluN1-N3A-N3B receptor with GluN1-N3A or GluN1-N3B receptors. **(H–J)** Model of GluN1-N3A, GluN1-N3B, and GluN1-N3A-N3B receptor transmembrane domain. The GluN1 subunits were transparent for clarify. View of a solvent-accessible surface carved along the pore axis using the MOLE.

In the GluN1-N3A receptor, the COM distance between GluN1(A) and GluN3A(C) in the R1–R1 interaction is 34.6 Å (Figures 5A, B), which is slightly larger than that in GluN1-N2A (PDB: 7EOS) (data not shown). Numerous amino acids stabilize the R1–R1 interaction through hydrophobic interactions. Specifically, the carbonyl group of D176 in GluN3A forms a hydroxyl interaction with G310 in GluN1, and the carbonyl group of D210 in GluN3A forms a hydrogen bond with the hydroxyl group of Y109 in GluN1. Additionally, the interaction is also stabilized by a hydrogen bond between C419 (GluN3A) and N70 (GluN1) (Figures 5A, B). From the ATD to the LBD, the entire receptor undergoes an anti-clockwise rotation around the hypothetical axis. This rotation results in the separation of GluN3A from its original dimer partner and the formation of a new dimer with another subunit. Overall, the LBD retains a pseudo-tetrameric structure as a dimer-of-dimers. Unlike the ATD, this pseudo-tetramer is tightly packed. During dimer formation, hydrogen bonds are formed between K531 in GluN1 and F644 and T645 in GluN3A, and between K790 in GluN1 and H781 in GluN3A. Additionally, the hydrogen bond between E786 in GluN1 and Y805 and A869 in GluN3A also contributes to the stability of the dimer (Figures 5B, C).

The ATD dimer interaction in GluN1-N3B is similar to that in GluN1-N3A. S132, D130, E80, G310, and N70 in GluN1 form hydrogen bonds with G140, A141, R89, D83, and C323/G324 in GluN3B, respectively, to maintain the dimer (Figures 5D, E). At the LBD level, there are significant differences between GluN1-N3A and GluN1-N3B, as the dimer formed is more loosely packed, stabilized only by the hydrogen bond between E751 in GluN1 and K809 in GluN3B (Figures 5E, F).

For the GluN1-N3A-N3B trimer, the ATDs adopt the structures like those in GluN1-N3A and GluN1-N3B receptors, with RMSDs of 0.90 and 0.85, respectively (Figure 5G, I, II). At the LBD level, the GluN1-N3A dimer in tri-GluN1-N3A-N3B adopts that in the di-GluN1-N3A receptor configuration, resulting in a small RMSD of 0.48. Conversely, the GluN1-N3B dimer at this position differs significantly from that in the di-GluN1-N3B receptor configuration (RMSD 8.56), resembling more closely the GluN1-N3A LBD dimer (Figure 5G, III, IV).

At the TMD level, the narrowest region is the pore-loop, located between M2 and M3. In the GluN1-N3A receptor, the loop is composed of R730, T731, A732, and A733 of GluN3A, with the narrowest point at R730. In GluN1-N3B, this loop comprises R629, R630, T631, V632, and S633 of GluN3B, with the narrowest point at T631. As previously mentioned, the assembly of tri-GluN1-N3A-N3B differs from that of di-GluN1-N3A and di-GluN1-N3B, with GluN3A and GluN3B positioned adjacently. In this receptor, the loop consists of G729, R730, T731, A732, and A733 in GluN3A and R629, R630, T631, V632, S633, and S634 in GluN3B, with the narrowest points at T731 and T631 (Figures 5H–J).

### Prediction of agonist- and antagonist-bound GluN3 structures

In our ongoing research, we utilized RoseTTAFold All-Atom to analyze small-molecule binding sites. Due to computational limitations, we focused on the LBD and TMD of GluN3A and GluN3B. To verify whether this approach differs from using the full-length model, we aligned the apo status of GluN3A from RoseTTAFold All-Atom prediction with the corresponding parts of the AlphaFold predicted full-length structures. The results showed very small RMSD values, indicating minimal differences.

**FIGURE 6:**
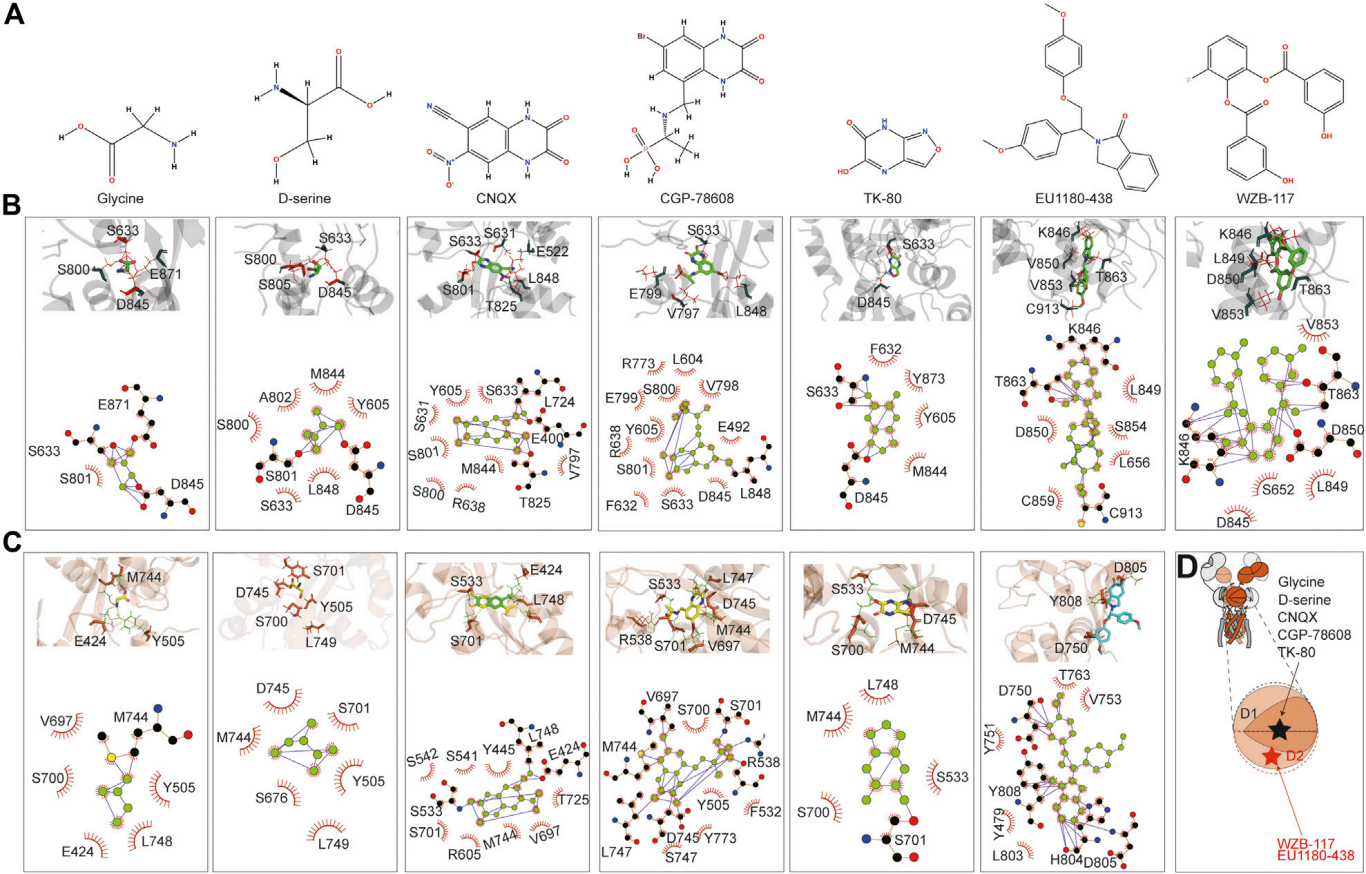
Prediction of the binding of agonist and antagonist. **(A)** Chemical structure of small molecules. **(B, C)** Model illustration and LigPlot of small-molecule binding interfaces of GluN3A and GluN3B, respectively. **(D)** Topology and model showing the binding site of small molecules.

The small molecules tested include the agonist glycine, inhibitors d-serine (Chatterton et al., 2002), CNQX (Madry et al., 2007), CGP-78608 (Otsu et al., 2019), TK80 (Kvist et al., 2013a), EU1180-438 (Zhu et al., 2020), and WZB-117 (Zeng et al., 2022). glycine, d-serine, CNQX, CGP-78608, and TK80 bound at the D1–D2 interface of the LBD, where the predicted glycine binding pocket is, and its vicinity. In contrast, EU1180-438 and WZB-117 bound to the inside of the D2 lobe (Figures 6A, D). We first examined glycine binding in GluN1-N3A and GluN1-N3B. LigPlot^+^ (Wallace et al., 1995) analysis revealed that in the GluN1-N3A receptor, S633, D845, and E871 form hydrogen bonds with glycine, with S801 making hydrophobic contacts. In the GluN1-N3B receptor, M744 formed a hydrogen bond with glycine, while several other amino acids, such as E424, Y505, V697, S700, and L748, contributed through hydrophobic contacts. S801 and D845 formed hydrogen bonds with d-serine in GluN1-N3A, stabilized by hydrophobic contacts from Y605, S633, S800, A802, M844, and L848. In GluN3B, unlike GluN3A, d-serine can be stabilized by only hydrophobic interactions with six amino acids: Y505, S676, S701, M744, D745, and L749.

CNQX formed hydrogen bonds with E400, L724, and T825 in GluN3A, while E424, S533, and L748 did so in GluN3B. CGP binding in GluN3A involved hydrogen bonding with L848, whereas in GluN3B, it was stabilized by hydrogen bonds with R538, V697, M744, D745, and L747. TK80 bound to GluN3A through hydrogen bonds with S633 and D845 and to GluN3B through S701.

EU1180-438 bound at a different location from the above agonists and antagonists, occupying the interior of the D2 lobe, as shown in Figure 6B. It interacts with K846, T863, and C913 in GluN3A and with D750, H804, D805, and Y808 in GluN3B. Previously, our work suggested that WZB-117 inhibits GluN1-N3A (Zeng et al., 2022). RoseTTAFold All-Atom predictions showed that WZB-117 binds near the site of EU1180-438, forming hydrogen bonds with K846, T863, and D850 in GluN3A (Figures 6B, C).

The predictions confirmed that the D1–D2 interface of the LBD is a critical binding site for both agonists (glycine and d-serine) and antagonists (CNQX, CGP-78608, and TK80). Glycine and d-serine binding sites were characterized by specific hydrogen bonds and hydrophobic interactions in both GluN3A and GluN3B receptors. However, there were distinct differences in the residues involved, reflecting the unique binding environments of each subunit. These interactions are crucial for understanding how different ligands stabilize the receptor and can inform future drug design efforts. Our predictions also provide a comprehensive map of potential drug targets. Specifically, the detailed interaction data can guide the design of new molecules with improved efficacy and specificity for these receptors. In summary, the RoseTTAFold All-Atom predictions provide valuable insights into the small-molecule binding mechanisms of GluN3A and GluN3B and highlight the specific interactions of various ligands, showcasing potential new avenues for therapeutic intervention targeting these receptor subunits.

## Discussion

In this study, we explored the expression and cell types of the GluN3 receptor in the brain via searching the single-cell sequence database and verified them in the brain and cultured neurons using immunofluorescence techniques. By applying AlphaFold2 and AlphaFold3, we predicted the structure of GluN3 containing NMDARs and compared them with cryo-EM resolved GluN1-N3A and GluN1-GluN2 receptors. Additionally, using the Rosetta-All-Atom algorithm, we predicted the binding of these receptors with small molecules and highlighted the binding pockets and interacting amino acids. Our results elucidate the structural characteristics of functional glycine-activated NMDARs, which are significantly expressed in the brain, providing a foundation for further understanding the structural and functional diversity of NMDARs.

NMDARs play crucial roles in the brain, including neurogenesis, brain development and synaptic plasticity (Larsen et al., 2011; Zeng et al., 2019), cognition and learning, memory and sleep (Roberts et al., 2009; Liu et al., 2016), and weight control (Petersen et al., 2024). Abnormal expression and function of these receptors are involved in many neurological and psychiatric diseases (Mohn et al., 1999), including stroke (Yan et al., 2020), Alzheimer’s disease (AD) (Cisse et al., 2011), Parkinson’s disease (PD) (Sharma et al., 2023), depression (Suzuki et al., 2017), and drug addiction (Yang et al., 2022). Much structural knowledge has been gained on conventional GluN1-N2 types of NMDARs (Karakas et al., 2011; Lee et al., 2014; Tajima et al., 2016; Zhu et al., 2016; Jalali-Yazdi et al., 2018; Chou et al., 2020), and although with slight differences, all di- or tri-NMDARs shared similar structural features. They formed as a bouquet-like shape with the longer side perpendicular to the cell membrane. Receptor subunits are arranged in an N1–N2–N1–N2 fashion with two-fold symmetry. The receptor domains are organized into three layers with the clamshell ATD layer on the top, the clamshell LBD layer in the middle, and the TMD layer at the bottom. Upon agonist binding, the LBD bi-lobes clamshell close, and then tension in the LBD–TMD linker increases the distance between the gating-ring residues that force the gate open. The functional and structural commonalities, including those described above, make the NMDARs important drug targets. Some of the drugs (Egunlusi and Joubert, 2024) failed in clinical trials, and those that were marketed often come with side effects. Beyond the limitations of the drugs, the diversity of different NMDAR subtypes may also contribute to these issues.

Nature is parsimonious, and each receptor subunit and subtype likely has its own functions. Traditional research has mostly focused on GluN2-containing NMDARs, with less attention being given to GluN3-containing NMDARs due to their later discovery and lower expression levels. Additionally, effective methods to distinguish them from traditional NMDARs have been lacking. Encouragingly, functional studies using competitive inhibitors targeting GluN1 have greatly amplified the current of GluN1-N3A receptors (Madry et al., 2008; Pfisterer et al., 2020; Rouzbeh et al., 2023), making it possible to study these subunits and revealing that they are involved in higher brain functions such as aversive behavior, similar to traditional NMDARs. Functional breakthroughs have also sparked interest in their structural biology. Recently, Hiro Furukawa’s lab published the first full-length structure of the GluN1-N3A receptor, revealing the unique structural characteristics of this receptor (Michalski and Furukawa, 2024). From structural studies and our own experiments (PDB: 8JF7), we know that the GluN3A receptor subunit is highly flexible, which limits the resolution of certain parts of the receptor structure, such as the ATD and TMD. The GluN1-N3A structure revealed by cryo-EM and predicted by AlphaFold exhibits some heterogeneity. We believe that several factors may contribute to this phenomenon. First, the cryo-EM structures are in glycine-bound and CNQX-bound states. In contrast, the AlphaFold predictions do not account for full-length agonist or antagonist-bound receptors, potentially representing the apo state of the receptor. This difference in ligand-binding states could lead to the observed discrepancies. Second, the GluN3A subunit in the GluN1-N3A receptor exhibits high flexibility, as observed in our structure of GluN1-N2A-N3A (PDB: 8JF7). In the cryo-EM model, the ATD density of this receptor is very diffuse, indicating structural variability. These factors highlight the inherent challenges in predicting and modeling the structure of flexible and dynamic receptor subunits, underscoring the importance of integrating multiple structural and functional approaches to gain a comprehensive understanding of GluN3-containing NMDARs.

In recent years, advanced protein prediction tools such as AlphaFold, RoseTTAFold, and ESM fold have made *de novo* protein structure prediction possible. These algorithms use evolutionary analysis combined with the most rational predictions to convert sequence information into three-dimensional structures. AlphaFold2 employs a trunk module, which utilizes self-attention transformers to process input data consisting of the query sequence, templates, and MSA, and a structure module, which employs 3D rigid body frames to directly generate 3D structures for training components (Jumper et al., 2021; Evans et al., 2022; Ahdritz et al., 2024). RoseTTAFold (Baek et al., 2021; Baek et al., 2023) uses a three-track network to process sequence, distance, and coordinate information. Extensive training and validation by numerous research groups have demonstrated that the predicted structures from these algorithms have high credibility. For NMDARs, we predicted the GluN1-N2A structure and found it nearly identical to published structures. Since AlphaFold2, including the newly released AlphaFold3 (Abramson et al., 2024), does not yet support user-driven small-molecule predictions, we used RoseTTAFold for this part of the work. Aligning RoseTTAFold structures to those from AlphaFold2 revealed great similarity. Although large protein language models constitute a significant advancement in solving the problem of protein structure prediction from sequence, they are not the gold standard. Many predicted structures still require validation through traditional structural biology methods such as X-ray crystallography, cryo-EM, and biochemical or functional studies. These models also face similar limitations as traditional structure methods, providing structures only for a specific state.

In conclusion, we used single-cell sequencing and immunofluorescence methods to detect GluN3A expression in the brain, providing crucial information for understanding the spatiotemporal expression of GluN3 receptors and laying the groundwork for studying their function and subcellular expression. Additionally, we employed two state-of-the-art methods to predict the structures of GluN1-N3A, GluN1-N3B, and GluN1-N3A-N3B receptors and their small-molecule binding pockets. This information helps in better understanding the structural characteristics of GluN3 receptors and the structural diversity of NMDARs.

## Materials and methods

The animal study protocol was approved by the Institutional Animal Care and Use Committee of Shenzhen Second People’s Hospital (Approval number: 20240076).

### Cell transfection and Western blotting

The pcDNA3-based plasmids that encode the human GluN1 (Uniprot: Q05586.1), the human GluN3A (Uniprot: Q9R1M7.1) fused with strep tag, and human GluN3B (Uniprot: O60391.2) fused with flag tag are used for the HEK293S cell PEI (Sigma Aldrich) transfection. All site-directed mutagenesis procedures were performed on the wild-type plasmid using KOD-Fx DNA polymerase (Takara). Cells were harvested 24–48 h after transfection and resuspended in a buffer containing 20 mM Tris-HCl (pH 7.4), 150 mM NaCl, 1% lauryl maltose neopentyl glycol (LMNG), and a protease-inhibitor cocktail (Roche). After centrifugation at 15,000 g, the supernatant was subjected to SDS–polyacrylamide gel electrophoresis (10% or 16%) in the presence or absence of 100 mM DTT. The proteins were transferred to PVDF membranes. The membranes were blocked with TBST (20 mM Tris-HCl (pH 7.4), 150 mM NaCl, and 0.1% Tween-20 containing 10% milk and then incubated with mouse monoclonal antibodies against GluN1 (Abcam), Strep (Abcam), and Flag (Proteintech), followed by HRP-conjugated anti-mouse or anti-rabbit antibodies (Proteintech). Protein bands were detected by an ECL detection kit (Beyotime).

### Brain acquisition and brain section immunocytochemistry

The newborn mice were used on the second day post-birth. The brain section preparation and imaging were prepared as previously described (Mo et al., 2018; Lei et al., 2023). Mice were anesthetized and transcranially perfused with 0.9% saline solution followed by 4% paraformaldehyde dissolved in 0.1 M phosphate buffer (pH 7.4). The brains were removed and cryoprotected using a graded sucrose series (20% and 30%) until they sank. A freezing microtome was used to cut 30–60-μm sections for imaging. Brain slices were stained with GluN3A (#AGC-030, Alomone) or GluN3B (ab35677, Abcam) and mouse-anti-MAP2 (ab254143, Abcam) overnight at 4°C. Then, the slices were stained with anti-rabbit IgG-Alexa Fluor 488 (ab150077, Abcam) and anti-mouse IgG-Alexa Fluor 594 (ab150116, Abcam) and mounted by mounting medium (Vector Laboratories). Brain slices were imaged by the VS120 microscope at excitation and emission wavelengths of 488 and 519 nm and 591 and 618 nm, respectively.

### Hippocampal neuron culture and immunocytochemistry

The hippocampal neurons were cultured as previously described with minor modifications (Zhou et al., 2021). In brief, the hippocampus was obtained from the 16–18-day-old Sprague–Dawley rat embryos. After removal of blood vessels, pia mater, and cortex, the freshly dissected hippocampus was sectioned into small fragments and digested with 0.25% trypsin for 15–30 min at 37°C and then terminated by the addition of DMEM with 10% fetal bovine serum. Cells were plated at a density of 5 × 10^5^ cells/mL on poly-L-lysine-coated dishes in DMEM with 10% fetal bovine serum. The medium was changed to neurobasal containing 2% B27 supplement and was changed twice a week. The 14–21-day-old neurons were used. The cell labeling of GluN3 and PSD95 was performed as previously reported (Kou et al., 2019) with minor modifications. Briefly, cells cultured on the coverslip were washed twice with PBS and fixed with 4% paraformaldehyde for 5 min. After washing in PBS three times, cells were first incubated in a blocking solution (0.3% Triton X-100% and 10% fetal calf serum dissolved in PBS) for 1 h at 37°C. Then, cells were stained with mouse-anti-PSD95 (ab13552, Abcam) and rabbit-anti-GluN3A or rabbit-anti-GluN3B overnight at 4°C and then with anti-rabbit IgG-Alexa Fluor 488 and anti-mouse IgG-Alexa Fluor 594. Coverslips were mounted by the mounting medium (Vector Laboratories).

### Sc-RNA sequencing analysis and phylogenetic analysis

Single-cell RNA sequence (Sc-RNA seq) analysis was carried out using the publicly available online dataset (https://singlecell.broadinstitute.org/single_cell). The sequences Q05586, Q12879.1, Q13224.3, Q14957.3, Q15399.2, Q9R1M7.1, O60391.2, P42261.2, P42262.3, P42263.2, P48058.2, P39086.1, Q13002.1, Q13003.3, Q16099.2, Q16478.2, Q9ULK0.2, and O43424.2 were used for the phylogenetic analysis. MEGA7 (Kumar et al., 2016) was used for protein sequence alignment with default parameters, and the maximum likelihood (ML) method was used.

### NMDARs structure prediction

The full amino acid sequences of GluN1, GluN3A, and GluN3B were downloaded from UniProt with access numbers Q05586.1, Q9R1M7.1, and O60391.2. Structures of GluN1, GluN3A, and GluN3A subunits predicted by AlphaFold were accessed from the AlphaFold Protein Structure Database (https://alphafold.ebi.ac.uk/). The ColabFold version of AlphaFold2 and AlphaFold3 was used to predict the structures of GluN1-N3A, GluN1-N3B, and GluN1-N3A-N3B receptors. For AlphaFold2, no templates were used, and the other setting was set as follows: max_seq = 508, max_extra_seq = 2048, number of recycle = 2, number of models = 2, and use amber to relax. Four 4090 GPUs from Matpool (https://matpool.com/) were used to predict the structure of these structures. It took approximately 5 days to predict the structure of GluN1-N3A and then approximately 20 h to predict the structure of GluN1-N3B and GluN1-N3A-N3B. For small-molecule binding, we used both the local installed RoseTTAFold All-Atom (Krishna et al., 2024) and the online version (https://www.tamarind.bio/app). To reduce computational burden, we used G486-Q970 for GluN3A prediction and P391-T861 for GluN3B prediction. The SDF files of glycine, D-serine, CNQX, and CGP-78608 were downloaded from PubChem. The SDF files of EU1180-438, TK-80, and WZB117 were produced by ChemDraw (V22).

## Data Availability

The datasets presented in this study can be found in online repositories. The names of the repository/repositories and accession number(s) can be found in the article/supplementary material.

## References

[B1] Abramson J. Adler J. Dunger J. Evans R. Green T. Pritzel A. (2024). Accurate structure prediction of biomolecular interactions with alphafold 3. Nature 630, 493–500. 10.1038/s41586-024-07487-w 38718835 PMC11168924

[B2] Ahdritz G. Bouatta N. Floristean C. Kadyan S. Xia Q. Gerecke W. (2024). Openfold: Retraining alphafold2 yields new insights into its learning mechanisms and capacity for generalization. Nat. Methods. 10.1038/s41592-024-02272-z PMC11645889 38744917

[B3] Andersson O. Stenqvist A. Attersand A. von Euler G. (2001). Nucleotide sequence, genomic organization, and chromosomal localization of genes encoding the human nmda receptor subunits nr3a and nr3b. Genomics 78 (3), 178–184. 10.1006/geno.2001.6666 11735224

[B4] Baek M. Anishchenko I. Humphreys I. R. Cong Q. Baker D. DiMaio F. (2023). Efficient and accurate prediction of protein structure using rosettafold2.

[B5] Baek M. DiMaio F. Anishchenko I. Dauparas J. Ovchinnikov S. Lee G. R. (2021). Accurate prediction of protein structures and interactions using a three-track neural network. Science 373 (6557), 871–876. 10.1126/science.abj8754 34282049 PMC7612213

[B6] Berg L. K. Larsson M. Morland C. Gundersen V. (2013). Pre- and postsynaptic localization of nmda receptor subunits at hippocampal mossy fibre synapses. Neuroscience 230, 139–150. 10.1016/j.neuroscience.2012.10.061 23159309

[B7] Bhattacharya S. Khatri A. Swanger S. A. DiRaddo J. O. Yi F. Hansen K. B. (2018). Triheteromeric glun1/glun2a/glun2c nmdars with unique single-channel properties are the dominant receptor population in cerebellar granule cells. Neuron 99 (2), 315–328.e5. 10.1016/j.neuron.2018.06.010 30056832 PMC6090556

[B8] Bossi S. Dhanasobhon D. Ellis-Davies G. C. R. Frontera J. de Brito Van Velze M. Lourenço J. (2022). Glun3a excitatory glycine receptors control adult cortical and amygdalar circuits. Neuron 110, 2438–2454.e8. 10.1016/j.neuron.2022.05.016 35700736 PMC9365314

[B9] Chatterton J. E. Awobuluyi M. Premkumar L. S. Takahashi H. Talantova M. Shin Y. (2002). Excitatory glycine receptors containing the nr3 family of nmda receptor subunits. Nature 415 (6873), 793–798. 10.1038/nature715 11823786

[B10] Chou T. H. Tajima N. Romero-Hernandez A. Furukawa H. (2020). Structural basis of functional transitions in mammalian nmda receptors. Cell. 182 (2), 357–371.e13. 10.1016/j.cell.2020.05.052 32610085 PMC8278726

[B11] Ciabarra A. M. Sullivan J. M. Gahn L. G. Pecht G. Heinemann S. Sevarino K. A. (1995). Cloning and characterization of chi-1: a developmentally regulated member of a novel class of the ionotropic glutamate receptor family. J. Neurosci. official J. Soc. Neurosci. 15 (10), 6498–6508. 10.1523/JNEUROSCI.15-10-06498.1995 PMC6577996 7472412

[B12] Cisse M. Halabisky B. Harris J. Devidze N. Dubal D. B. Sun B. (2011). Reversing ephb2 depletion rescues cognitive functions in alzheimer model. Nature 469 (7328), 47–52. 10.1038/nature09635 21113149 PMC3030448

[B13] Egunlusi A. O. Joubert J. (2024). Nmda receptor antagonists: Emerging insights into molecular mechanisms and clinical applications in neurological disorders. Pharm. (Basel) 17 (5), 639. 10.3390/ph17050639 PMC11124131 38794209

[B14] Evans R. O’Neill M. Pritzel A. Antropova N. Senior A. Green T. (2022). Protein complex prediction with alphafold-multimer.

[B15] Hansen K. B. Ogden K. K. Yuan H. Traynelis S. F. (2014). Distinct functional and pharmacological properties of triheteromeric glun1/glun2a/glun2b nmda receptors. Neuron 81 (5), 1084–1096. 10.1016/j.neuron.2014.01.035 24607230 PMC3957490

[B16] Iacobucci G. J. Popescu G. K. (2017). Nmda receptors: Linking physiological output to biophysical operation. Nat. Rev. Neurosci. 18 (4), 236–249. 10.1038/nrn.2017.24 28303017 PMC5640446

[B17] Jalali-Yazdi F. Chowdhury S. Yoshioka C. Gouaux E. (2018). Mechanisms for zinc and proton inhibition of the glun1/glun2a nmda receptor. Cell. 175 (6), 1520–1532.e15. 10.1016/j.cell.2018.10.043 30500536 PMC6333211

[B18] Jumper J. Evans R. Pritzel A. Green T. Figurnov M. Ronneberger O. (2021). Highly accurate protein structure prediction with alphafold. Nature 596 (7873), 583–589. 10.1038/s41586-021-03819-2 34265844 PMC8371605

[B19] Karakas E. Simorowski N. Furukawa H. (2011). Subunit arrangement and phenylethanolamine binding in glun1/glun2b nmda receptors. Nature 475 (7355), 249–253. 10.1038/nature10180 21677647 PMC3171209

[B20] Kou Z. W. Mo J. L. Wu K. W. Qiu M. H. Huang Y. L. Tao F. (2019). Vascular endothelial growth factor increases the function of calcium-impermeable ampa receptor glua2 subunit in astrocytes via activation of protein kinase c signaling pathway. Glia 67 (7), 1344–1358. 10.1002/glia.23609 30883902 PMC6594043

[B21] KrishnaR.WangJ.AhernW.SturmfelsP.VenkateshP.KalvetI. (2024). Generalized biomolecular modeling and design with rosettafold all-atom. Science 384 (6693), eadl2528. 10.1126/science.adl2528 38452047

[B22] Kumar S. Stecher G. Tamura K. (2016). Mega7: molecular evolutionary genetics analysis version 7.0 for bigger datasets. Mol. Biol. Evol. 33 (7), 1870–1874. 10.1093/molbev/msw054 27004904 PMC8210823

[B23] Kvist T. Greenwood J. R. Hansen K. B. Traynelis S. F. Brauner-Osborne H. (2013a). Structure-based discovery of antagonists for glun3-containing n-methyl-d-aspartate receptors. Neuropharmacology 75, 324–336. 10.1016/j.neuropharm.2013.08.003 23973313 PMC3865070

[B24] Kvist T. Steffensen T. B. Greenwood J. R. Mehrzad Tabrizi F. Hansen K. B. Gajhede M. (2013b). Crystal structure and pharmacological characterization of a novel n-methyl-d-aspartate (nmda) receptor antagonist at the glun1 glycine binding site. J. Biol. Chem. 288 (46), 33124–33135. 10.1074/jbc.M113.480210 24072709 PMC3829161

[B25] Larsen R. S. Corlew R. J. Henson M. A. Roberts A. C. Mishina M. Watanabe M. (2011). Nr3a-containing nmdars promote neurotransmitter release and spike timing-dependent plasticity. Nat. Neurosci. 14 (3), 338–344. 10.1038/nn.2750 21297630 PMC3474337

[B26] Lee C. H. Lü W. Michel J. C. Goehring A. Du J. Song X. (2014). Nmda receptor structures reveal subunit arrangement and pore architecture. Nature 511 (7508), 191–197. 10.1038/nature13548 25008524 PMC4263351

[B27] Lei Y. Chen X. Mo J. L. Lv L. L. Kou Z. W. Sun F. Y. (2023). Vascular endothelial growth factor promotes transdifferentiation of astrocytes into neurons via activation of the mapk/erk-pax6 signal pathway. Glia 71 (7), 1648–1666. 10.1002/glia.24361 36960578

[B28] Lin Z. Akin H. Rao R. Hie B. Zhu Z. Lu W. (2023). Evolutionary-scale prediction of atomic-level protein structure with a language model. Science 379 (6637), 1123–1130. 10.1126/science.ade2574 36927031

[B29] Liu S. Liu Q. Tabuchi M. Wu M. N. (2016). Sleep drive is encoded by neural plastic changes in a dedicated circuit. Cell. 165 (6), 1347–1360. 10.1016/j.cell.2016.04.013 27212237 PMC4892967

[B30] Lu W. Du J. Goehring A. Gouaux E. (2017). Cryo-em structures of the triheteromeric nmda receptor and its allosteric modulation. Science 355 (6331), eaal3729. 10.1126/science.aal3729 28232581 PMC5568803

[B31] Madry C. Betz H. Geiger J. R. Laube B. (2008). Supralinear potentiation of nr1/nr3a excitatory glycine receptors by zn2+ and nr1 antagonist. Proc. Natl. Acad. Sci. U. S. A. 105 (34), 12563–12568. 10.1073/pnas.0805624105 18711142 PMC2527951

[B32] Madry C. Mesic I. Bartholomaus I. Nicke A. Betz H. Laube B. (2007). Principal role of nr3 subunits in nr1/nr3 excitatory glycine receptor function. Biochem. biophysical Res. Commun. 354 (1), 102–108. 10.1016/j.bbrc.2006.12.153 17214961

[B33] Mesic I. Madry C. Geider K. Bernhard M. Betz H. Laube B. (2016). The n-terminal domain of the glun3a subunit determines the efficacy of glycine-activated nmda receptors. Neuropharmacology 105, 133–141. 10.1016/j.neuropharm.2016.01.014 26777280

[B34] Michalski K. Furukawa H. (2024). Structure and function of glun1-3a nmda receptor excitatory glycine receptor channel. Sci. Adv. 10 (15), eadl5952. 10.1126/sciadv.adl5952 38598639 PMC11006217

[B35] Mirdita M. Schutze K. Moriwaki Y. Heo L. Ovchinnikov S. Steinegger M. (2022). Colabfold: making protein folding accessible to all. Nat. Methods 19 (6), 679–682. 10.1038/s41592-022-01488-1 35637307 PMC9184281

[B36] Mo J. L. Liu Q. Kou Z. W. Wu K. W. Yang P. Chen X. H. (2018). Microrna-365 modulates astrocyte conversion into neuron in adult rat brain after stroke by targeting pax6. Glia 66 (7), 1346–1362. 10.1002/glia.23308 29451327 PMC6001668

[B37] Mohn A. R. Gainetdinov R. R. Caron M. G. Koller B. H. (1999). Mice with reduced nmda receptor expression display behaviors related to schizophrenia. Cell. 98 (4), 427–436. 10.1016/s0092-8674(00)81972-8 10481908

[B38] Murillo A. Navarro A. I. Puelles E. Zhang Y. Petros T. J. Perez-Otano I. (2021). Temporal dynamics and neuronal specificity of grin3a expression in the mouse forebrain. Cereb. cortex 31 (4), 1914–1926. 10.1093/cercor/bhaa330 33290502 PMC7945027

[B39] Otsu Y. Darcq E. Pietrajtis K. Matyas F. Schwartz E. Bessaih T. (2019). Control of aversion by glycine-gated glun1/glun3a nmda receptors in the adult medial habenula. Science 366 (6462), 250–254. 10.1126/science.aax1522 31601771 PMC7556698

[B40] Paoletti P. Bellone C. Zhou Q. (2013). Nmda receptor subunit diversity: impact on receptor properties, synaptic plasticity and disease. Nat. Rev. Neurosci. 14 (6), 383–400. 10.1038/nrn3504 23686171

[B41] Perez-Otano I. Larsen R. S. Wesseling J. F. (2016). Emerging roles of glun3-containing nmda receptors in the cns. Nat. Rev. Neurosci. 17 (10), 623–635. 10.1038/nrn.2016.92 27558536

[B42] Petersen J. Ludwig M. Q. Juozaityte V. Ranea-Robles P. Svendsen C. Hwang E. (2024). Glp-1-directed nmda receptor antagonism for obesity treatment. Nature 629, 1133–1141. 10.1038/s41586-024-07419-8 38750368 PMC11136670

[B43] Pfisterer U. Petukhov V. Demharter S. Meichsner J. Thompson J. J. Batiuk M. Y. (2020). Identification of epilepsy-associated neuronal subtypes and gene expression underlying epileptogenesis. Nat. Commun. 11 (1), 5038. 10.1038/s41467-020-18752-7 33028830 PMC7541486

[B44] Rauner C. Kohr G. (2011). Triheteromeric nr1/nr2a/nr2b receptors constitute the major n-methyl-d-aspartate receptor population in adult hippocampal synapses. J. Biol. Chem. 286 (9), 7558–7566. 10.1074/jbc.M110.182600 21190942 PMC3045010

[B45] Roberts A. C. Diez-Garcia J. Rodriguiz R. M. Lopez I. P. Lujan R. Martinez-Turrillas R. (2009). Downregulation of nr3a-containing nmdars is required for synapse maturation and memory consolidation. Neuron 63 (3), 342–356. 10.1016/j.neuron.2009.06.016 19679074 PMC3448958

[B46] Rouzbeh N. Rau A. R. Benton A. J. Yi F. Anderson C. M. Johns M. R. (2023). Allosteric modulation of glun1/glun3 nmda receptors by glun1-selective competitive antagonists. J. general physiology 155 (6), e202313340. 10.1085/jgp.202313340 PMC10125900 37078900

[B47] Sharma R. Neupane C. Pham T. L. Lee M. Lee S. Lee S. Y. (2023). Tonic activation of nr2d-containing nmdars exacerbates dopaminergic neuronal loss in mptp-injected parkinsonian mice. J. Neurosci. official J. Soc. Neurosci. 43 (46), 7730–7744. 10.1523/JNEUROSCI.1955-22.2023 PMC10648527 37726169

[B48] Stroebel D. Paoletti P. (2021). Architecture and function of nmda receptors: an evolutionary perspective. J. physiology 599 (10), 2615–2638. 10.1113/JP279028 32786006

[B49] Suzuki K. Nosyreva E. Hunt K. W. Kavalali E. T. Monteggia L. M. (2017). Effects of a ketamine metabolite on synaptic nmdar function. Nature 546 (7659), E1–E3. 10.1038/nature22084 28640258

[B50] Tajima N. Karakas E. Grant T. Simorowski N. Diaz-Avalos R. Grigorieff N. (2016). Activation of nmda receptors and the mechanism of inhibition by ifenprodil. Nature 534 (7605), 63–68. 10.1038/nature17679 27135925 PMC5136294

[B51] Traynelis S. F. Wollmuth L. P. McBain C. J. Menniti F. S. Vance K. M. Ogden K. K. (2010). Glutamate receptor ion channels: structure, regulation, and function. Pharmacol. Rev. 62 (3), 405–496. 10.1124/pr.109.002451 20716669 PMC2964903

[B52] Wallace A. C. Laskowski R. A. Thornton J. M. (1995). Ligplot: a program to generate schematic diagrams of protein-ligand interactions. Protein Eng. 8 (2), 127–134. 10.1093/protein/8.2.127 7630882

[B53] Wang H. Lv S. Stroebel D. Zhang J. Pan Y. Huang X. (2021). Gating mechanism and a modulatory niche of human glun1-glun2a nmda receptors. Neuron 109 (15), 2443–2456.e5. 10.1016/j.neuron.2021.05.031 34186027

[B54] Wang J. X. Furukawa H. (2019). Dissecting diverse functions of nmda receptors by structural biology. Curr. Opin. Struct. Biol. 54, 34–42. 10.1016/j.sbi.2018.12.009 30703613 PMC6592722

[B55] Wee K. S. Tan F. C. Cheong Y. P. Khanna S. Low C. M. (2016). Ontogenic profile and synaptic distribution of glun3 proteins in the rat brain and hippocampal neurons. Neurochem. Res. 41 (1-2), 290–297. 10.1007/s11064-015-1794-8 26700428

[B56] Yan J. Bengtson C. P. Buchthal B. Hagenston A. M. Bading H. (2020). Coupling of nmda receptors and trpm4 guides discovery of unconventional neuroprotectants. Science 370 (6513), eaay3302. 10.1126/science.aay3302 33033186

[B57] Yang H. J. Hempel B. J. Bi G. H. He Y. Zhang H. Y. Gardner E. L. (2022). Elevation of extracellular glutamate by blockade of astrocyte glutamate transporters inhibits cocaine reinforcement in rats via a nmda-glun2b receptor mechanism. J. Neurosci. official J. Soc. Neurosci. 42 (11), 2327–2343. 10.1523/JNEUROSCI.1432-21.2022 PMC8936610 35091501

[B58] Yi F. Bhattacharya S. Thompson C. M. Traynelis S. F. Hansen K. B. (2019). Functional and pharmacological properties of triheteromeric glun1/2b/2d nmda receptors. J. physiology 597, 5495–5514. 10.1113/JP278168 PMC6858497 31541561

[B59] Zeng Q. Michael I. P. Zhang P. Saghafinia S. Knott G. Jiao W. (2019). Synaptic proximity enables nmdar signalling to promote brain metastasis. Nature 573 (7775), 526–531. 10.1038/s41586-019-1576-6 31534217 PMC6837873

[B60] Zeng Y. Zheng Y. Zhang T. Ye F. Zhan L. Kou Z. (2022). Identification of a subtype-selective allosteric inhibitor of glun1/glun3 nmda receptors. Front. Pharmacol. 13, 888308. 10.3389/fphar.2022.888308 35754487 PMC9218946

[B61] Zhou D. Xie C. Li X. Song N. Kou Z. Zhang T. (2021). Rare presence of autoantibodies targeting to nmda and gabaa receptors in schizophrenia patients. Schizophr. Res. 249, 93–97. 10.1016/j.schres.2021.12.002 34916095

[B62] Zhu S. Stein R. A. Yoshioka C. Lee C. H. Goehring A. McHaourab H. S. (2016). Mechanism of nmda receptor inhibition and activation. Cell. 165 (3), 704–714. 10.1016/j.cell.2016.03.028 27062927 PMC4914038

[B63] Zhu Z. Yi F. Epplin M. P. Liu D. Summer S. L. Mizu R. (2020). Negative allosteric modulation of glun1/glun3 nmda receptors. Neuropharmacology 176, 108117. 10.1016/j.neuropharm.2020.108117 32389749 PMC7530031

